# Current and Future Challenges for Rehabilitation for Inflammatory Arthritis

**DOI:** 10.3390/jcm13061808

**Published:** 2024-03-21

**Authors:** Rikke Helene Moe, Thea P. M. Vliet Vlieland

**Affiliations:** 1 Center for Treatment of Rheumatic and Musculoskeletal Diseases (REMEDY), Diakonhjemmet Hospital, P.O. Box 23, Vinderen, No-0319 Oslo, Norway; 2 Department of Orthopaedics, Rehabilitation and Physical Therapy, Leiden University Center, Albinusdreef 2, 2333 ZA Leiden, The Netherlands; t.p.m.vliet_vlieland@lumc.nl; 3 Basalt Rehabilitation, Vrederustlaan 180, 2543 SW The Hague, The Netherlands; 4 Faculty of Healthcare, University of Applied Sciences, Zernikedreef 1, 2333 CK Leiden, The Netherlands

**Keywords:** rehabilitation, rheumatology, inflammatory arthritis, rheumatoid arthritis, spondyloarthritis, rheumatic and musculoskeletal diseases

## Abstract

This narrative review discusses the importance of rehabilitation in rheumatic and musculoskeletal diseases (RMDs), ultimately aiming to reduce their impact on individuals and society. It specifically emphasizes the need for rehabilitation in inflammatory arthritis (IA), particularly in cases where medical management is insufficient. It acknowledges that the complexity of rehabilitation demands a flexible approach. Thereby, it touches on the various models of rehabilitation, which may include multidisciplinary team care, extended practice models, shared care, remote care, and work rehabilitation. It discusses the challenges in research, practice, and policy implementation. In research, the need for innovative research designs is highlighted, whereas regarding clinical practice the importance of early detection of disability and patient engagement is underlined, as well as the role of telehealth and AI in reshaping the rehabilitation landscape. Financial barriers and work force shortages are identified as challenges that hinder the effective delivery of rehabilitative care. On the policy level, this paper suggests that the allocation of healthcare resources often prioritizes acute conditions over chronic diseases, leading to disparities in care. This paper concludes by emphasizing the critical role of evidence-based rehabilitation in improving the quality of life for people with RMDs, in particular for those with IA, and promoting their healthy aging. It also calls for tailored rehabilitation models and the early identification of persons with rehabilitation needs as future challenges in this field.

## 1. Introduction

Scientists are presently engaged in the pursuit of discovering a cure for rheumatic and musculoskeletal diseases (RMDs), the largest cause of physical disability in Europe, accounting for about 50% of years lived with disabilities (YLDs) [1]. Given the substantial impact of RMDs on individuals and the society [1,2], there is a need to address gaps in knowledge and to develop guidelines and practice recommendations for the management of long-term RMD-related disability, including the extra challenges regarding healthy ageing [3]. Inflammatory RMDs, such as rheumatoid arthritis (RA) or axial spondyloarthritis (axSpA), are relatively common RMDs contributing considerably to the general burden of RMDs. In particular, the subgroups of patients who do not respond optimally to medical management may need rehabilitation. It was found that in RA, 5 to 20% of persons with RA fulfill the difficult-to-treat criteria (D2T) [4,5,6,7], whereas persisting disease activity is associated with functional limitations and decreased quality of life.

Currently, most professional recommendations on the management of people with inflammatory arthritis and disability include, apart from medical care, access to a multidisciplinary team [8]. In inflammatory RMDs, the multidisciplinary team usually includes, in addition to the medical care provided by physicians, nurses, clinical nurse specialists or nurse practitioners, physical therapists, occupational therapists, psychologists, social workers, podiatrists, dieticians, and others [9]. While the required competences of team members have been extensively documented for rheumatologists [10], the core competences for other health professionals involved in the management of people with RMDs have been formulated only relatively recently [11].

Traditionally, rehabilitation for people with inflammatory RMDs has been provided in the form of multidisciplinary team care, delivered in an inpatient, day patient, or outpatient setting, either in general or specialized hospitals or rehabilitation centers [9]. Although such admissions for multidisciplinary team care have been relatively common in this patient group, the evidence for their effectiveness is lacking. Regarding RA, a systematic review published in 2016 identified 10 randomized controlled trials (RCTs) with the possibility to pool data from five studies [12]. That review concluded that there is limited evidence for the effectiveness of a multidisciplinary team care on disability, disease activity, or quality of life [12]. Since that review, a limited number of (observational) studies have been published. In axSpA, the evidence is even more scarce, as most research is either focused on one type of intervention, in particular exercise, or is concerned with spa therapy or balneotherapy. One of the exceptions is a study concluding on positive effects of a multidisciplinary intervention on disease activity, pain, function, and wellbeing after 3 weeks of inpatient treatment [13]. Apart from RCTs, a number of uncontrolled studies on the effectiveness of rehabilitation in people with RMDs, including inflammatory arthritis (IA), have been performed, in general reporting beneficial effects between admission, discharge, and/or follow up [14]. The Scandinavian Team Arthritis Register—European Team Initiative for Care Research (STAR-ETIC), a collaborative effort involving Danish, Dutch, Norwegian, and Swedish researchers and clinicians dedicated to the rehabilitation of individuals with IA established consensus on the collection of data from 18 distinct institutions at admission and discharge [15]. The registry encompassed over 700 patients with IA, of whom 67–91% reported experiencing one or more co-morbidities. Rehabilitation interventions demonstrated a positive impact on health-related quality of life, particularly among individuals with more severe disease consequences. Notably, after adjusting for possible confounders, those reporting worse function, lower psychological wellbeing, and more pain or more fatigue were likely to have a larger change in HRQoL after team rehabilitation [16]. More recently, the initial findings from an extensive multicenter longitudinal rehabilitation cohort in Norway, known as RehabNytte, involved the recruitment of nearly 4000 participants, with the predominant diagnostic group being people with RMDs. The results indicate a consistent improvement in work ability and function over the 12-month post-admission period. Notably, actively participating in the formulation of one’s own rehabilitation goals nearly tripled the likelihood of successfully achieving those goals [17,18].

For many years, the provision of traditional multidisciplinary team care was more or less self-evident and amply available in most European countries [19,20]. Over the past decades, there have, however, been many developments in the treatment of people with RMDs as well as health care and society. These developments include, but are not limited to, more effective medical treatment of IA, in particular the following: the evolving role of people with RMDs, the lack of resources for the delivery of multidisciplinary treatment (personnel, facilities, costs), the shortage of specialized health professionals in the workforce, and the increasing availability of remote care technologies (telehealth). In line with these developments, there is a need to re-think the current and future delivery of multidisciplinary team care for people with IA [21,22].

This narrative review addresses the complexity of rehabilitation for people with inflammatory RMDs, examples of various models of delivery of rehabilitative care, and describes a number of challenges for future research in this area.

## 2. Complexity of Rehabilitation

Rehabilitation interventions concur with the definition of complex interventions, stating that an intervention might be considered complex because of the following: the properties of the intervention itself, such as the number of components involved; the range of behaviors targeted; the expertise and skills required by those delivering and receiving the intervention; the number of groups, settings, or levels targeted; or the permitted level of flexibility of the intervention or its components [23]. Rehabilitation for people with inflammatory RMDs should, however, not be simply regarded as a complex intervention in rheumatology, it should be considered as a concept [24,25], a multifaceted, dynamic domain of healthcare. Each rehabilitation trajectory is unique, with the actual content, dosage, and mode of delivery being dependent on numerous factors. First of all, the rehabilitative approach has to be aligned with the specific problems patients are facing due to their particular RMD. As an example, patients with RA predominantly experience involvement of the peripheral joints, commonly resulting in hand disability, whereas in patients with axSpA the spine is mainly affected, leading to problems related to long periods of standing and active movement of the spine. But apart from the consequences of an individual’s specific RMD and comorbidities, personal factors including motivation and expectations, and heterogeneity due to social support, resource disparities, care location, and geographic variations must be considered as well. A universal framework capturing the complexity of health and disability is offered by the World Health Organization’s International Classification of Functioning, Disability and Health (ICF) [26], describing functioning in terms of “Body Functions and Structure” (e.g., pain, fatigue) [27] or “Activities and Participation” (e.g., work ability) [28] and its interrelationships with personal and contextual factors. Given the complex and dynamic nature of health and disability as well as the concept of rehabilitation, thinking in terms of models of care rather than very specific interventions is warranted [21].

## 3. Models of Rehabilitation for People with Inflammatory Arthritis

Apart from the traditional multidisciplinary team care provided in an inpatient, outpatient, or day patient care setting, a number of other models of care to provide integrated care to people with IA have been developed and evaluated. Rehabilitation systems and practice vary greatly between countries, but the most common models of care have been identified in previous reviews of the literature. In addition to the already available overviews [21,22], we searched the literature for more recent systematic reviews on non-traditional models of care in people with IA. Subsequently, we identified the most relevant individual clinical studies from the available reviews or published after their closing date. The results of this exploration are summarized below.

### 3.1. Extended and Advanced Scope of Practice

Although extended and advanced scope of practice models may mainly concern rheumatologists and nurses or physical therapist and are focused on particular aspects of care such as drug monitoring and prescriptions or triage, there are also examples of models where other professionals are involved [21,22]. One example is the primary therapist model, where physical therapists and occupational therapists function as case managers and multi-skilled rehabilitation professionals [29,30]. Primary therapists may consult their respective physical or occupational therapist colleague, rather than transferring the patient for completion of the treatment. Overall, it was concluded that primary therapist care was associated with better outcomes (pain, function, knowledge, utility) than traditional care, but at higher costs. Here it must be noted that the outcomes of the economic analysis may be dependent on the costs of physical and occupational therapy in a specific country, whereas the applicability of the model on the national level depends on various factors, such as the availability of those professionals and their educational level or the reimbursement policies for their services.

### 3.2. Shared Care Model

With shared care models, regular patient follow up is provided by non-physician care providers and/or primary care physicians rather than specialists, with patient-initiated follow up in specialty care, including the multidisciplinary team, as needed [31]. Overall, with shared care models, equivalent outcomes compared to usual care (disease activity, radiographic damage, quality of life) are obtained, at similar or lower costs and similar or higher patient satisfaction. However, the selection of patients for whom this model is appropriate is a matter of debate in the light of health equity. Recently, based on the principles of value-based health care [32,33], the implementation of such a model has been proposed in Norway [34]. As the provision of shared care demands both synchronous and asynchronous methods of communication between health care professionals and people with RMDs, the availability of remote care technology, or telehealth, becomes all the more important.

### 3.3. Remote Care (Telehealth) Models

Rehabilitation is witnessing a shift towards remote and home-based care options, offering patients access to care directly from their homes, often integrating wearable devices and real-time needs-based communication, intervention, monitoring, assessment, and interventions increasing accessibility of rehabilitation services [35,36]. In a recent systematic review encompassing 14 clinical trials on remote care applications in inflammatory arthritis it was found that in the included studies remote monitoring predominated as a prevalent modality, primarily conducted via telephone or video calls [37,38]. Notably, remote care was widely perceived as user friendly and efficient, delivering favorable outcomes in terms of time and cost savings. In contrast, an umbrella review targeting people in need of rehabilitation including people with RMDs focusing on studies on remote care to encourage physical activity found that there is a paucity of high-quality research-based evidence favoring one or another specific technology, device, or tool to support remote rehabilitation, such as wearables, apps, and trackers [39]. In general, it must also be noted that, so far, most research has only been applied to a selection of people willing to take part in testing this kind of innovative rehabilitation interventions. It was also found that their implementation may be hampered by legal and financial barriers [37,38,39,40,41].

### 3.4. Work Rehabilitation Models

People with IA face more work-related challenges than the general population. They have a greater likelihood of unemployment, absenteeism (work absence), and presenteeism (not fully productive). Approximately one-third of individuals with inflammatory arthropathies leave their employment within three years of diagnosis, a figure that rises to 50% within a decade [42,43]. Rehabilitation can help facilitate work ability and participation, and job loss prevention may have a positive effect on work ability, absenteeism, and job loss among persons with IA [44]. Known prognostic factors reported to contribute to work disability in rheumatoid arthritis are older age, low education, high physical job demands, low functional capacity, and sickness absence [45,46]. A fundamental objective of rehabilitation programs for individuals of working age is to promote the maintenance of paid employment or reintegration to the workforce. However, when considering the inherent diversity among recipients of rehabilitation services, it becomes apparent that, thus far, these interventions have yielded a positive, but relatively modest impact on work participation [28,47]. For the feasibility of work rehabilitation models in a specific country, it must be noted that the delivery is dependent on the availability of and access to health professionals involved, such as rehabilitation specialists or occupational therapists. Herewith, not only the health care system but also the social security system must be taken into account, with the role of occupational health services attached to companies varying between countries. Such variation limits the generalizability of the work rehabilitation interventions developed and proven to be successful on the national level.

## 4. Challenges for Research

Over the past 20 years, rehabilitation for people with IA has changed dramatically, mainly due to successful medical options. Meanwhile, the overarching goal of helping people with IA optimize their biopsychosocial functioning, participation, and to achieve the best possible quality of life has remained the same. The goals, content, and focus of rehabilitation have shifted focus from the basic activities of daily life to more complex activities that must be performed in order to meet the demands of and fully engage in contemporary society.

### 4.1. Clinical Trial Design

As most studies on the effectiveness of rehabilitation were carried out decades ago, the evidence for the most effective rehabilitation strategies being able to meet the current demands of people with IA and limitations in activities and participation is scarce. One of the challenges for the conduct of randomized clinical trials in rehabilitation is allocation concealment. For ethical reasons, all participants must be fully informed about the study design and the specific contents of the intervention and control conditions. An alternative study design is the Zelen design, where random assignment of treatment is performed prior to patient or participant consent [48]. Following randomization, a participant would receive information and asked to consent to the assigned treatment [48]. However, such a design may not be accepted by institutional research review boards. While the blinding of patients and clinicians is thus impossible in most cases, the blinding of assessors is a possibility, provided that patients and clinicians are well instructed not to disclose the treatment allocation at the assessments.

Another challenge is regarding the description of the often comprehensive and complex treatments. Specifically, for this purpose, a framework is available: the Template for Intervention Description and Replication (TIDIER) [49]. This template may, in addition to the MRC Framework for developing and evaluating complex interventions [23], be helpful in the phases where the rehabilitation interventions are designed and/or executed.

It must also be noted that, as previously mentioned, the rehabilitation concept in IA is commonly based on individual goals and treatment plans agreed upon by means of a shared decision process in collaboration between the member(s) of the multidisciplinary team and the individual patient. This flexible approach has consequences for the selection of the study design, the development of the intervention, and selection of outcomes when it comes to demonstration of the effectiveness or cost effectiveness of rehabilitation trials. Given the complexity of the intervention, its delivery may be, more than with single interventions, subject to developments among stakeholders and the context. The need for flexibility is acknowledged by experts on research on complex interventions in health care [23]. However, conventional RCTs do not usually offer much flexibility; thus, alternative research designs allowing the researcher to respond to developments related to the intervention or the context along the way must be considered. Alternative designs like interrupted series designs may be able to capture individual change [50], and long-term follow-up studies may help us to determine the efficacy of more complex and individually tailored interventions within the framework of the characteristics of minimizing the individual and societal burden of life with IA [51]. In addition, patient-specific outcome measures, reflecting the severity of the problems that are most bothersome for the individual patients, are needed. Examples of these are the McMaster Toronto Arthritis Patient Preference Disability Questionnaire (Mactar) [52], the Canadian Occupational Performance Measure (COPM) [53], and the Patient-Specific Functional Scale (PSFS) [54].

Overall, due to the abovementioned challenges, clinical trials in rehabilitation are prone to the introduction of various sources of bias [55]. A number of bias domains, including blinding, can result in either underestimation or overestimation of treatment effects, although the precise impact of bias in RCTs in rehabilitation is difficult to determine [55]. Nevertheless, it is very much recommended for researchers in rehabilitation to adhere to the appropriate guidelines for this particular research area, in particular the Consolidated Standards of Reporting Trials (CONSORT) Extension for nonpharmacological trials [56,57] and the previously mentioned TIDIER template [49].

### 4.2. Implementation

A known implementation challenge is the time needed for the implementation process, in addition to other factors like knowledge, allocated personnel, and finances [58,59]. The process can be sped up by employing hybrid implementation-effectiveness study designs [60]. If such a model is used, implementation outcomes are gathered during the conduct of the clinical trial and the results can be used for the development of a tailored implementation strategy.

## 5. Discussion

### 5.1. Early Detection of Disability

The current practice of medical treatment of people with inflammatory RMDs is very much focused on monitoring and evaluation of disease activity. Thereby, limitations in daily functioning or participation restrictions, that can, apart from disease activity, be related to personal and environmental factors, may thus remain unrecognized. Therefore, there is a need for the development and evaluation of strategies aimed at the early detection of functional disability, its diagnosis and access to appropriate rehabilitation services, as well as on secondary prevention strategies focusing on specific goals for people who can and want to adhere to lifestyle advice and help those who can and want to stay in work [61,62,63,64]. Regarding the latter, additional education for health professionals delivering lifestyle interventions is likely to be needed, as supporting behavioral changes in patients requires specific professional skills.

Effectively managing inflammatory rheumatic diseases in a long-term perspective requires both flexible systems that allow individually tailored care and timely comprehensive biopsychosocial team approaches. This was recently addressed in a mixed methods study from the United Kingdom, including persons with RA and their healthcare professionals [65]. Findings indicated two pathways to address the most common disease management challenges: the “early care package” initiated around the time of diagnosis, introducing the members of the multidisciplinary team and the possibilities of influencing life with RA, and an additional tailored care package for people with established disease, addressing clinical, psychological, and social needs across disease management stages, with the aim of engaging the right health professional adjusted to specific needs [65].

### 5.2. Patient Engagement

Fostering equality via strengthening patient engagement is a focus area for the European Commission [66] and is a field under development. Patient engagement can help improve future research and service provision, help shape models of rehabilitation, and enable equal opportunities [67,68,69].

### 5.3. Financial Barriers

The societal burden of IA is, among other factors, affected by the high costs of treatment and productivity loss. Current value-based health care models [32,33] may serve as a barrier to rehabilitation, as the role of rehabilitation for IA may not merely be to improve symptoms and disability, but often to learn how to live their best possible lives despite their disease. A known challenge in the reimbursement of these kinds of interventions, as many countries have a widely implemented marked economic health care system, may be that in-person visits often result in more activity-based financing. The implementation of health care funding policy has shown contrasting results on the possible improvement of efficiency. Adapting the financial systems and funding mechanisms to meet current and future rehabilitation options and needs is warranted to deliver more efficient care [70,71]. Also, the financing of rehabilitation frequently relates to diagnoses and specific treatments, rather than being aligned with considerations of overall functioning and health [72]. Many countries are also in a situation where the integration of rehabilitation in the health financing systems are limited; this often results in small budgets for rehabilitation that do not meet the need of the population. This also has the tendency of increasing inequality of care, as people in need of rehabilitation must spend more of their private economical resources or private insurance to cover the costs, leading to inaccessibility of rehabilitation for those who need it the most. This theme is currently addressed via the Rehabilitation 2030 initiative by the WHO [3,73].

### 5.4. Policy Priorities

Governments and health care systems often prioritize acute conditions from which there is a more immediate risk of death over chronic diseases with increased mortality rates after long-term disability [2]. This dilemma is reflected in the different main theoretical aspects behind these different care models, where the classical reductionistic biomedical aspects meet the holistic biopsychosocial aspects to compete for the same resources [74,75]. A recent example affecting people with RMDs was the COVID-19 pandemic. Allocation of health resources to combat the virus resulted in reallocation of resources and health care specialists during the pandemic, with a long-term effect of losing specialists to other fields of specialty [76]. An additional challenge pertains to the supplementary obstacles posed by silo-based healthcare systems for people in need of rehabilitation. These people frequently encounter the necessity to navigate across multiple healthcare services to address their diverse needs, potentially resulting in the receipt of high-quality care for only specific components of their complex health conditions [72].

### 5.5. WHO Rehabilitation Packages

To ease the implementation of the core elements to be included when the different countries are planning and budgeting for their future rehabilitation services and specialist work force, the WHO has launched packages of interventions for musculoskeletal diseases, and other health conditions available at https://www.who.int/publications/i/item/9789240071100 (accessed on 11 March 2024).

## 6. Conclusions

The aim of this narrative review was to describe the complexity of rehabilitation, with several alternative models for its delivery for inflammatory arthritis as examples, and the challenges for future research, delivery of care, and health policy. Rehabilitation plays a pivotal role in the continuum of healthcare, addressing the multifaceted needs of individuals with disabilities or chronic health conditions. Recent advancements in healthcare technologies, particularly remote care and AI, are quickly reshaping the rehabilitation landscape. Numerous contemporary reports attest to the collaborative efforts between researchers, clinicians, and technology firms directed at enhancing the trajectory of remote care (telehealth) for future advancements. By embracing evidence-based rehabilitation interventions and technological advancements, healthcare can shape a future where rehabilitation is more accessible, personalized, and impactful in improving the lives of persons with diverse healthcare needs. Rehabilitation plays a pivotal role in enhancing the quality of life for people affected by IA, fostering a reduction in disability and, consequently, contributing to healthy aging.

## 7. Future Directions

Rehabilitation holds an immense promise, particularly in addressing the substantial personal and societal burden associated with IA. However, the traditional multidisciplinary team care model is no longer feasible in many countries or in the longer term. There is thus a significant need for the development and evaluation of rehabilitative services that offer the same quality of care while applying other modes of delivery. As a prime source of new models of care, innovation is crucial in reshaping rehabilitation approaches and assessments. Examples of innovative elements include greater involvement of patients in the monitoring of their levels of ability and participation and taking the appropriate steps in the case of deterioration. Identifying the most appropriate monitoring instruments, defining cut-off values, and setting up care pathways for specific problems, as well as selecting the most appropriate modes of delivery tailored to each individual patient are topics that need to be addressed to further develop this field. The increasing involvement of patients with RMDs in such developments is crucial [77].

Another important field of development is the implementation of guidelines and recommendations for the management of RMDs among the health professionals involved in care. This is essential to ensure that every patient with an RMD receives high-quality care. There are numerous guidelines and recommendations available regarding rehabilitation or integrated care for people with RMDs [78], but their uptake is limited. Given the large variation in the organization of and access to rehabilitation between different countries, it is likely that tailored implementation strategies adapted to the national situation are needed on a country-by-country basis.

Novel opportunities are found in initiatives fostering innovation within the rehabilitation domain, with a specific focus on the implementation of evidence-based practices. Targeting disability, in particularly for people with D2T arthritis, promoting healthy aging, and incorporating early detection as a preventive strategy are critical components that should guide future research and practice in the field of rehabilitation.

## Data Availability

Not applicable.
